# Metatranscriptome analysis reveals bacterial symbiont contributions to lower termite physiology and potential immune functions

**DOI:** 10.1186/s12864-016-3126-z

**Published:** 2016-10-01

**Authors:** Brittany F. Peterson, Michael E. Scharf

**Affiliations:** 1 Department of Entomology, Purdue University, 901 W. State St, West Lafayette, IN 47907-2089 USA; 2 Present address: Center for Insect Science, University of Arizona, 1007 E. Lowell St, Tucson, AZ 85721 USA

**Keywords:** Termite, Metatranscriptome, Symbiosis, Microbial ecology, Ribo-depletion

## Abstract

**Background:**

Symbioses throughout the animal kingdom are known to extend physiological and ecological capabilities to hosts. Insect-microbe associations are extremely common and are often related to novel niche exploitation, fitness advantages, and even speciation events. These phenomena include expansions in host diet, detoxification of insecticides and toxins, and increased defense against pathogens. However, dissecting the contributions of individual groups of symbionts at the molecular level is often underexplored due to methodological and analytical limitations. Termites are one of the best studied systems for physiological collaborations between host and symbiota; however, most work in lower termites (those with bacterial and protist symbionts) focuses on the eukaryotic members of this symbiotic consortium. Here we present a metatranscriptomic analysis which provides novel insights into bacterial contributions to the holobiont of the eastern subterranean termite, *Reticulitermes flavipes,* in the presence and absence of a fungal pathogen.

**Results:**

Using a customized ribodepletion strategy, a metatranscriptome assembly was obtained representing the host termite as well as bacterial and protist symbiota. Sequence data provide new insights into biosynthesis, catabolism, and transport of major organic molecules and ions by the gut consortium, and corroborate previous findings suggesting that bacteria play direct roles in nitrogen fixation, amino acid biosynthesis, and lignocellulose digestion. With regard to fungal pathogen challenge, a total of 563 differentially expressed candidate host and symbiont contigs were identified (162 up- and 401 downregulated; α/FDR = 0.05) including an upregulated bacterial amidohydrolase.

**Conclusions:**

This study presents the most complete bacterial metatranscriptome from a lower termite and provides a framework on which to build a more complete model of termite-symbiont interactions including, but not limited to, digestion and pathogen defense.

**Electronic supplementary material:**

The online version of this article (doi:10.1186/s12864-016-3126-z) contains supplementary material, which is available to authorized users.

## Background

The intimate association between termites and microbes is so tightly linked that often what it means to be a termite cannot be discussed without describing their symbiotic relationships. In the 1920’s L.R. Cleveland described this association and the necessity of these ‘parasites’ to termite survival [9, 10]. The discovery of protist-produced cellulases in lower termites as a means to thrive on their nitrogen poor, recalcitrant wood diet forever solidified termite symbiosis as the quintessential example of insect-microbe collaboration [40]. For decades, termites were thought to rely entirely on symbiota for the digestion of their food until a highly-active, highly-expressed, endogenous β-1, 4-endoglucanase was identified in a lower termite species [42]. This finding shifted the perspective of this symbiosis from unidirectional to collaborative. As tools in molecular biology advanced, more and more cellulytic enzymes were identified from the symbiotic partners and hosts in all termite symbioses [12, 26, 36, 38, 47, 48]. The synergy demonstrated by these enzymes from lower termites begins to explain how the efficiency of this system led to the broad success of termites [32].

Termite research consistently addresses digestive symbioses, but lower termite literature has almost entirely focused on protist-termite collaborations. Until recently, bacterial contributions to wood digestion in lower termites were largely disregarded [2]. However, bacteria are now known to play important roles in nitrogen cycling, hemicellulose and aromatic compound degradation, and acetate metabolism which likely contribute to the maintenance of efficient cellulose digestion in the termite gut [15, 16, 22, 36]. For example, reductions in bacterial number and diversity after antimicrobial drug feeding have been linked to reductions in lignocellulytic activity in the termite gut [28].

Though the traditional, intuitive role for gut bacteria may be nutritional, gut microbiota can have profound impacts on their insect hosts. For example, symbionts of stinkbugs have been shown to confer pesticide resistance to their hosts, Colorado potato beetles circumvent plant defenses with help from bacterial symbionts, and microbes appear to be at least partially responsible for crop-rotation resistance in the western corn rootworm [7, 8, 21]. These examples suggest that insect-associated microbes play more diverse roles than previously thought. Indeed, lower termite symbionts have recently been credited with contributing important anti-fungal enzymes which extend pathogen defense to their insect hosts, and in particular, beta-1, 3-glucanases from protist symbionts have been identified as a source of fungal deactivation in lower termites [31]. However, while symbiotic actinobacteria have been shown to provide anti-fungal functions within the nest walls of some subterranean termites [6] and bacteria play important roles in termite and ant fungus-farming mutualisms [1, 41], there has been no mechanistic link between gut bacteria and pathogen defense in lower termites. It is thus reasonable to postulate that lower termite-associated gut bacteria are contributing to host physiology in more ways than just nutrition/digestion.

This idea of “collaborative physiology” represents a joint effort by the members of the holobiont to accomplish basic physiological tasks, like digestion and immunity. Steps in assessing the extent and mechanisms of these collaborations require approaches which encompass the entire micro-ecosystem that is the termite gut. Assessing the holobiont allows for a more complete picture of functional capacity of individual members of the consortium but also sheds light on interspecific collaborations [27].

In the present study we harnessed the power of next-generation sequencing to explore the contributions and potential collaborations of the termite host and its hindgut consortium. The aims of this research were two-fold: 1) to describe bacterial contributions to the gut metatranscriptome and 2) to identify potential mechanisms of bacterial-derived anti-fungal defense against the fungal pathogen, *Beauveria bassiana*. Using a ribo-depletive library preparation strategy, we captured a holobiont metatranscriptome that included the often-overlooked prokaryotic symbiota. Our findings highlight the potential for extensive collaboration between symbiota and the host termite including an abundance of transcripts encoding bacterial nutrient and metabolite transporters, amino acid synthetic enzymes, and carbohydrate metabolism. Additionally, we identified differentially expressed transcripts between fungal infected and uninfected termites, as well as a candidate amidohydrolase mechanism for bacterially-mediated anti-fungal activity in the termite gut. Overall, this study provides a snapshot of the potential functions of bacteria in *R. flavipes* guts and begins to shed light on the extent to which host-prokaryote collaboration plays a role in defense against fungal entomopathogens.

## Methods

### Termites and pathogen rearing

*R. flavipes* termites used in this project were collected on the Purdue University campus in West Lafayette, IN between May and July 2014. Colonies were reared in darkness at 22 ± 2 °C with ~40 % relative humidity and provided with pine wood shims and brown paper towels as a food source. The pathogen used in this study was *B. bassiana* isolate #5477 which was cultured in darkness on PDA at room temperature. To collect conidia for bioassays, 10 to 12-day old *B. bassiana* culture plates were flooded with 5 mL of filter sterilized 0.5 % Tween-20. Conidial concentration was determined via hemocytometer and diluted to 1.25 × 10^4^ conidia/mL for termite inoculation.

### Bioassay setup & dissections

Groups of 30 termite workers were either submerged in a suspension of 1.25 × 10^4^ conidia/mL in 0.5 % filter sterilized Tween-20 or Tween-20 alone for one minute. Termites were transferred to 33 mm plastic dishes containing 2 cm^2^ of Whatman filter paper wetted with 100 μL of deionized water. Bioassays were held at 28 ± 2 °C for 48 h in complete darkness in a growth chamber. Paired treatment groups (control and pathogen challenged) from 3 distinct termite colonies served as biological replicates, for a total of 6 samples. After 48 h in bioassay, termite whole guts were dissected from 25 workers per treatment, submerged in RNAlater (ThermoFisher Scientific) and stored at −80 °C.

### RNA isolation & library preparation

Samples were thawed on ice and all RNAlater aspirated leaving only termite gut tissue. This tissue was then homogenized in Promega SV Total RNA Isolation Kit lysis buffer and the manufacturer’s protocol followed to extract total RNA from all samples. RNA concentration and relative purity was quantified using a NanoDrop 2000 spectrophotometer (ThermoFisher Scientific).

To reduce eukaryotic bias in library preparation, total RNA was ribodepleted rather than enriched for mRNA. The metatranscriptome libraries were prepared from total RNA using the Ovation Complete Prokaryotic RNA-Seq DR Multiplex System 1–8 (NuGEN) with the addition of custom oligos targeting the 18S rRNA from eukaryotic species anticipated to be in the samples (termite, protists, and fungal pathogen; Additional file 1: Table S1). Total RNA (250 ng) was used as starting material for the Ovation kit and cDNA was synthesized following manufacturer instructions. After second strand synthesis, cDNA samples were frozen overnight at −20 °C.

After thawing, samples were sonicated using a Covaris E210 in Covaris #520045 6 × 16 mm microtubes using the parameters specified in the Ovation protocol (Intensity = 5 not 5 %) and transferred into fresh 200uL microtubes and stored overnight at −20 °C. cDNA purification, end repair, barcode ligation, first strand selection, and first strand purification were performed per manufacturer’s protocol. At the strand selection II step, 1 μL of the 100 μM custom oligo mix was added to 16 μL of Solution SS5 to deplete eukaryotic rRNAs from samples. Adapter cleavage was performed as specified in the kit manual. Library amplification master mix was made fresh per protocol instructions, but the thermocycler program was modified from the manufacturer’s protocol as follows: 95 °C for 2 min, 20 cycles of 95 °C for 30s, 60 °C for 90s, and 65 °C for 5 min. Bead purification of the amplified library was done with a multi-channel pipet to minimize incubation bias of the libraries. Each sample type (control or fungal pathogen challenged) and biological rep (1–3) were indexed separately for a total of 6 prepped libraries for sequencing.

### Metatranscriptome sequencing, assembly, annotation, and analysis

**Fig. 1:**
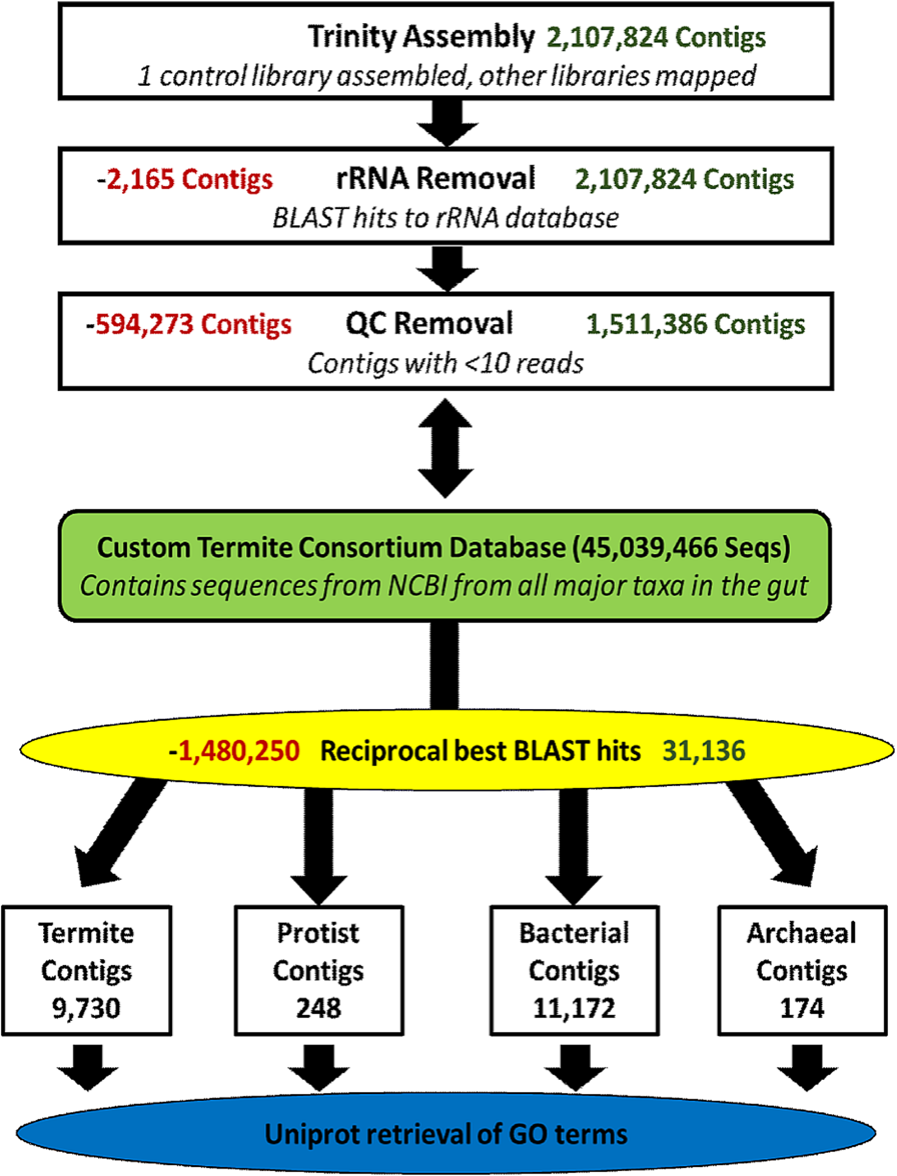
Workflow of metatranscriptome annotation. Red numbers on the left at various steps represent the number of contigs removed during that step and green numbers on the right represent the number of contigs exiting the step

A workflow summarizing the major steps in the metatranscriptome analysis is shown in Fig. 1. Purified, indexed libraries were submitted to the PUGC facility for quality control screening and sequenced in 1 lane on the Illumina HiSeq2500 platform to produce 2 × 100 paired-end reads. Contigs of one control library containing the least rRNA reads were assembled *de novo* using Trinity 2.1.1 by PUGC; all other libraries were mapped to this assembly to produce a read count table. Any contigs with less than 10 reads across samples or identified by homology search as rRNA were filtered out.

In an effort to annotate the contigs in a taxon-specific way, a custom termite consortium database was built from existing data in the NCBI refseq protein database (bacteria, archaea, select protists, Table 1) and the *Zootermopsis nevadensis* OGS, as this is the only lower termite genome available at the present time [37]. Reciprocal best hits (RBHs) were determined by using BLAST to identify best hits with our assembly as the query and the custom termite consortium database as the subject (BLASTx) and vice versa (tBLASTn). Contigs that were RBHs with entries in this database at an e-value 1e-5, or less, were carried through for additional analysis. This ensured a conservative annotation of contigs in this assembly, although this also limited detection of novel and redundant genes within the consortium.

**Table 1:** Summary table of custom termite consortium database

Composition of custom termite consortium database		
Group	Source	No. of Seqs.
Archaea	NCBI Archaea RefSeq	851,375
Bacteria	NCBI Bacteria RefSeq	44,100,533
Protist	NCBI RefSeq for Parabasalia, Oxymonadida, Diplomonadida, and Gregarinasina	72,948
Termite	OGS for *Zootermopsis nevadensis*	14,610
	Total sequences	45,039,466

Database was constructed to annotate the metatranscriptome in a taxon specific manner. Archaeal, bacterial, and protist sequences were obtained from the NCBI RefSeq database and termite sequences were obtained from the *Zootermopsis nevadensis* official gene set (OGS)

To associate contigs with GO terms, the Genbank identifiers from the list of RBH for each taxon group (bacteria, archaea, protists, and termite) were analyzed using the Uniprot retrieve/ID mapping function (www.uniprot.org/uploadlists/). Using the Bioconductor package in R statistical software, edgeR differential expression analyses were done on read counts for all contigs to detect responses to the fungal pathogen (α and FDR = 0.05). To determine if any biological processes or molecular functions were enriched in a taxon group, lists of GO terms from each taxon group (bacteria, archaea, protists, or termite) were compared to all GO terms in the gene set and enrichment was determined with a two-sided Fisher’s exact test using the topGO function in the Bioconductor package.

### Metatranscriptome validation

qPCR was used as an independent validation of read count values used to generate contigs for differential expression analysis. Contigs representing termite, bacteria, protist, up-regulated, down-regulated, and no change groups were selected for qPCR validation (Additional file 1: Table S2). Using the cDNA samples generated as described previously, 1 μL of cDNA, 1 μL each of contig-specific forward/reverse primers, 7 μL nuclease-free water, and 10 μL of SensiFast SYBR no ROX master mix (Bioline) were combined for qPCR using a Bio-Rad CFX-96 system. After an initial denaturation step (10 min. at 95 °C), 45 cycles of denaturing (30 s. at 95 °C), annealing (30 s. at 50 °C), and extension (30 s. at 72 °C) were performed with a real-time scan of fluorescence taken after each cycle. The log ratio CT values were regressed against log ratio of metatranscriptome counts per million values as a measure of congruency. Regression data were analyzed by the Spearman correlation method.

### Post-hoc assays of reactive oxygen species abundance, glutathione S-transferase activity, and amidohydrolase expression

To further validate metatranscriptome findings, additional biochemical assays were performed to test the potential for oxidative stress and increased antioxidant enzyme activity in the termite gut following *B. bassiana* challenge. Reactive oxygen species (ROS) were detected using a modified FOX1A assay [11]. Bioassays were repeated on new termites and guts dissected as described above. A mixture of 100 μL of termite gut homogenate containing 10 termite gut equivalents in 100 mM sodium phosphate buffer was combined with 100 μL nanopure water and 100 μL of FOX1A reagent and then incubated for 40 min in the dark. Endpoint absorbance was measured at 580 nm and compared to a hydrogen peroxide standard curve to estimate reactive oxygen species concentration. This was repeated in triplicate for control and pathogen-challenged groups and all biological replicates.

GST activity was measured kinetically using CDNB as the substrate. Freshly prepared 1 mM CDNB in 100 mM sodium phosphate buffer (pH 7.0) was combined with 10 μl of gut homogenate with or without 5 mM reduced L-glutathione for a total reaction volume of 235 μl. Absorbance was read kinetically for 10 min and mean velocity for all samples were calculated. The mean velocity of glutathione-plus samples was blank-corrected with their corresponding glutathione-minus controls. Specific activity was calculated using the extinction coefficient of CDNB of 9.5 mM^-1^ cm^-1^.

Both ROS and GST assays measurements were normalized per milligram of protein in each sample. Protein concentration of samples was estimated using the Pierce Coomassie Plus Bradford Assay Kit (Thermo-Fisher Scientific).

To investigate whether symbiont removal reduced amidohydrolase expression, groups of 10 workers from three independent colonies were subjected to a treatment of 5 % kanamycin (controls received water only) for 48-h and subsequently challenged with a low dose of *B. bassiana* conidia (as above). 5 % Kanamycin was used for bacterial symbiont removal based on extensive preliminary research [28]. These bioassays were held for 48-h before termite guts were dissected for RNA extraction. cDNA was synthesized using the Bioline SensiFast cDNA Synthesis kit using 1 μg total RNA as template. qPCR was performed using the SensiFast no ROX SYBR Master Mix (Bioline) and amidohydrolase 2 gene specific primers as well as Actin 5C as a reference gene (Additional file 1: Table S2).

Similarly, post-hoc bioassays were performed to determine if 5 % kanamycin treatment was sufficient to increase termite susceptibility to *B. bassiana*. As above, groups of 10 termites were treated with 5 % kanamycin or water (controls) for 48-h, and then inoculated with a low dose of *B. bassiana* conidia suspended in 0.5 % Tween-20 or a blank of 0.5 % Tween-20 solution (controls). All together, these four groups allowed us to account for any baseline mortality caused by antibiotic treatment. These bioassays were held for 7 days before scoring survivorship.

## Results

### Ribodepletion effectively removes rRNA from the hyper-diverse termite gut

**Fig. 2:**
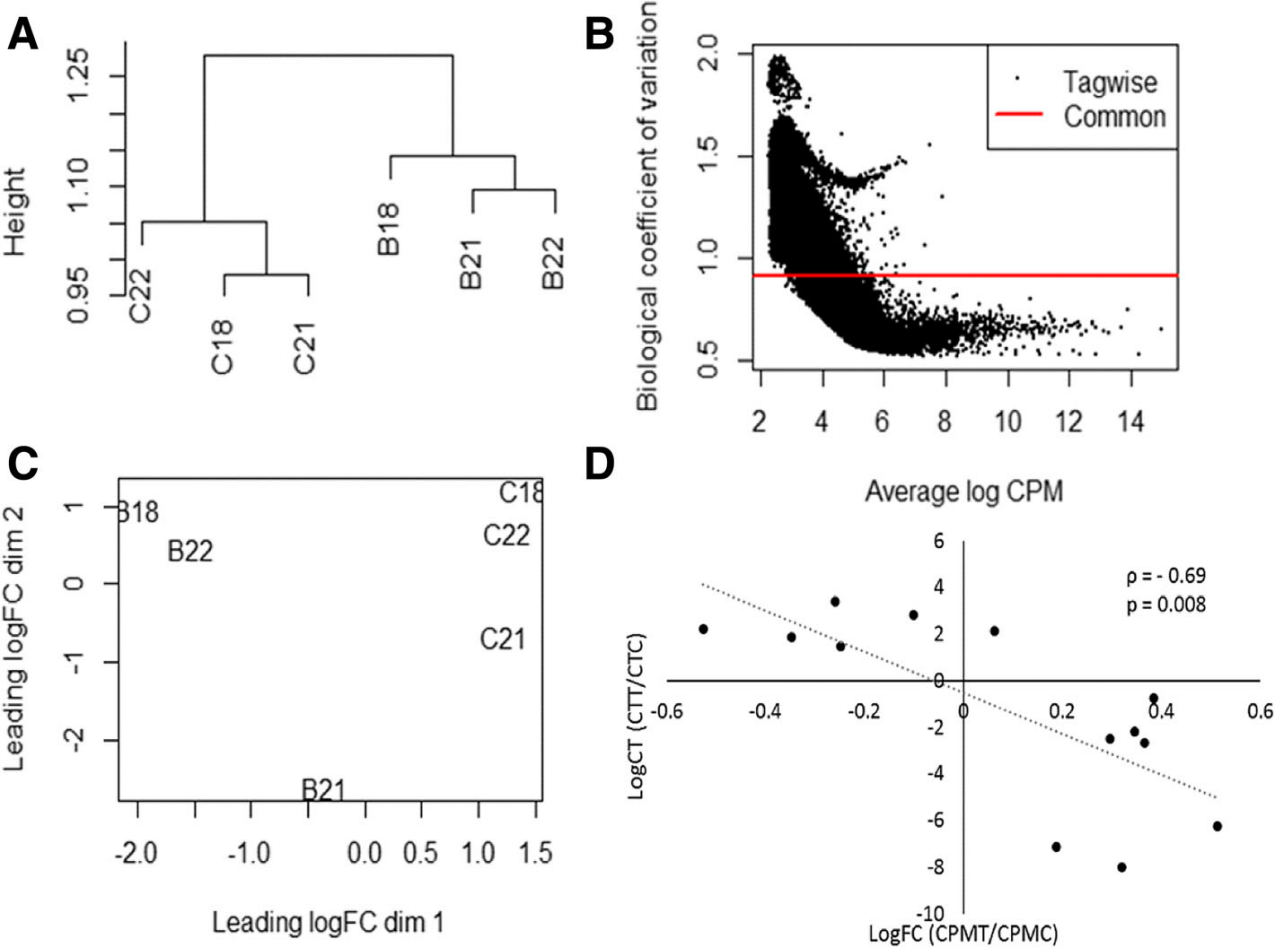
Quality control analyses of the metatranscriptome sequence. **a** Cluster dendrogram based on a Pearson distribution of all contigs following normalization. Samples are labelled with letters indicating their treatment (C = control, B = *Beauveria)* and colony number. **b** Plot of Biological coefficient of variation vs. average logCPM. Each spot represents an individual contig. **c** Multiple dimension scaling plot showing distances in gene expression profiles across biological replicates and treatment groups. **d** Results of a validation experiment showing the correlation between logCT from qPCR analyses (CT of treatment/CT of control) vs. logFC (counts per million of treatment/counts per million of control). Spearman’s correlation coefficient rho (ρ) reported shows a significant, strong negative correlation (*p* = 0.008). Each data point represents a single gene (*n* = 14; See Additional file 1: Table S2)

In total, 2,107,824 contigs were assembled *de novo* from a control termite gut holotranscriptome (Table 2B). The analyzed sequences contained 1.2 % rRNA reads and the average rRNA contamination across all samples was estimated to be 12.33 % (Table 2A). It should be noted that pathogen challenged samples had lower read count numbers and higher rRNA than that of the control samples. This may be due to inefficiency in ribodepletion for fungal rRNAs. Of the assembled contigs 258,251 had an N_50_ length of 652 bases and average length of 704 bases (Table 2B). After filtering out rRNA and contigs with <10 reads across samples 1,511,386 contigs remained. Additionally, a cluster dendrogram based on a Pearson distribution and a multiple dimension scaling plot both agree in that samples cluster together based on treatment type (control vs. *B. bassiana* challenge) rather than by termite colony (colonies nos. 18, 21, or 22) (Fig. 2a, c).

**Table 2:** Summary of sequencing and assembly statistics

A	Sequencing statistics		
	*Sample*	*# Reads*	*rRNA*
	C18	97,357,292	3.0 %
	C21	96,954,778	6.3 %
	C22*	95,291,086	1.2 %
	B18	75,834,616	21.5 %
	B21	83,141,808	14.9 %
	B22	58,954,982	27.3 %
B	Assembly statistics		
	*All*	No. Contigs	2,107,824
		N50	356
		Avg. Length	361
	*>500 bases*	No. Contigs	258,251
		N50	652
		Avg. Length	704

A) Summary of sequencing statistics. * Indicates the library used for Trinity assembly which was selected because of low rRNA contamination. B) Summary of *de novo* Trinity assembly. Samples are labelled with a letter indicating their treatment (C = control, B = *Beauveria)* and colony number

### Summary of the holotranscriptome

**Fig. 3:**
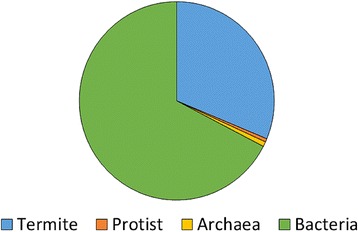
Pie chart demonstrating taxon distributions for annotated contigs. Total proportions of the contigs from the metatranscriptome were annotated as belonging to each taxonomic group. Only those contigs having reciprocal blast hits (RBH) are included

In order to assign annotations to potential genes of interest, all assembled, filtered contigs were reciprocally BLASTed to determine putative function and taxonomic assignment (Fig. 1). A total of 31,156 contigs had RBHs with entries in the custom termite consortium database with an e-value of 1e-5 or less. Each of these annotations was associated with a taxonomic group: termite, protist, bacteria, or archaea (Fig. 3). Of these, 21,269 contigs had hits in the Uniprot ID matching database. It should be noted that the protist and archaeal annotations are more incomplete due to a lack of information available in the NCBI RefSeq database regarding termite symbiont groups (archaea, bacteria, and specific protists). The bacterial and termite contigs, however, are considerably more complete with annotations in many critical biosynthetic, catabolic, transport, and stress response processes (Table 3). Additionally, some of these categories, like amino acid biosynthesis for example, appear to have signatures of complementation between the host termite and bacterial symbionts (Table 3).

**Table 3:** Summary of select putative bacterial and termite contig functions

Functional annotations of contigs			
Category		Bacterial	Termite
Biosynthesis			
	*Amino Acid*	^a^ *143*	*11*
	Alanine	2	0
	Arginine	15	0
	Asparagine	3	1
	Cysteine	3	0
	Glutamine	2	1
	Glycine	2	1
	Histidine	28	0
	Isoleucine	7	0
	Leucine	6	0
	Lysine	16	0
	Methionine	18	3
	Phenylalanine	2	0
	Proline	9	1
	Pyrrolysine	1	0
	Serine	5	1
	Threonine	5	0
	Tryptophan	6	0
	Tyrosine	3	0
	Valine	5	0
	Other	22	3
	*Vitamin*	*29*	*0*
	Thiamine	27	0
	B6	2	0
	*Fatty Acid*	*33*	*12*
	*Lipid*	*5*	*2*
	*Cellular Structure*	*50*	*8*
	Phospholipid	16	8
	Peptidoglycan	34	0
Metabolism			
	*Carbohydrate*	*276*	*88*
	Glycosyl Hydrolases	204	34
	Glycosyl Transferase	67	39
	Polysaccharide Lyase	3	7
	Carbohydrate Esterase	2	8
	*Chitin*	*2*	*33*
	*Nitrogen*	*32*	*7*
	Amidohydrolase	14	3
	Nitrogenase	6	0
	Nitroreductase	9	0
	Urease	9	0
	Other	8	4
	*Protein*	^a^ *207*	^a^ *228*
	Aminopeptidases	15	9
	Aspartic-type Peptidases	6	9
	Carboxypeptidases	18	30
	Cysteine-type Peptidases	12	24
	Dipeptidase	11	12
	Metallopeptidases	43	61
	Serine-type Peptidases	76	81
	Threonine-type Peptidases	1	12
	Other Peptidases	31	19
Transport			
	*ABC Transporters*	*355*	*2*
	Amino Acid	27	0
	Urea	4	0
	Carbohydrate	42	0
	Metal Ion	26	0
	C4-dicarboxylate	5	0
	Multidrug	13	0
	Excinuclease	6	0
	Other	232	2
	*Other MFS Transporters*	*37*	*11*
	*Other Transporters*	*163*	*191*
Stress regulation			
	*Antioxidant/Detoxification Enzymes*	*69*	*71*
	Aldo/Keto Reductase	7	1
	Alkyl Hydroperoxide Reductase	8	0
	Catalase	1	1
	Cytochrome Oxidase P450s	0	31
	Desulfoferrodoxin	4	0
	Ferredoxin	27	0
	Glutaredoxin	0	3
	Glutathione Peroxidase	1	2
	Glutathione S-Transferase	5	6
	Peroxidase	0	6
	Peroxidasin	0	3
	Superoxide Dismutase	0	3
	Thioredoxin	7	14
	Other	9	1
	*Chaperonin*	*15*	*16*
	*Other*	*3*	*4*

Bacterial and termite contig putative functions based on reciprocal best hits and GO molecular function. ^a^Some candidates possess multi-functional annotations

### Differential gene expression analysis

Using edgeR analysis, a total of 563 contigs exhibited significant differential expression in response to fungal pathogen challenge (FDR *p* < 0.05). The majority of these contigs were annotated as host-origin, but some symbiont contigs were also impacted (Table 4). In total, 162 contigs were up-regulated and 401 were down-regulated (Table 4). Of the differentially expressed contigs, only 223 contained Uniprot ID matches and 225 had annotated GO terms. Relative expression observed in the metatranscriptome was validated by qPCR. Log CT ratios were correlated to Log CPM ratios between treatment and control samples (Fig. 2d). Spearman’s correlation coefficient rho (ρ = −0.69) shows a significant negative correlation (*p* = 0.008), as would be anticipated.

**Table 4:** Summary of differentially expressed contigs

Summary statistics table for metatranscriptome RNAseq			
Taxon	Number up-regulated	No change	Number down-regulated
Termite	134	9339	258
Protist	18	228	2
Bacteria	10	20,852	141
Archaea	0	174	0
Total	162	30,593	401

Differentially expressed contigs from each taxon based on RBH annotations. Significantly up- and down-regulated contigs from each taxon were determined at α/FDR = 0.05

In general, the termite contigs up-regulated in response to *B. bassiana* challenge reveal the hallmarks of oxidative stress (Table 5). Thirty-one ribosomal proteins were up-regulated which has been associated with slowed or inhibited protein translation. Additionally, a mitochondrial peroxiredoxin and a GST were up-regulated 2.8-fold and 5.1-fold respectively. Stress and immune-associated 10 kDa heat shock protein and ferritin were up-regulated as well. Calcium (Ca^2+^), iron (Fe^3+^), zinc (Zn^2+^), and other generic metal ion binding GO terms were abundant in the pathogen up-regulated termite contigs. Also notably, several components of the OXPHOS pathway were up-regulated (subunits of complex I, complex III, and complex IV and cytochrome c), however; ATP synthase contigs were not differentially expressed.

**Table 5:** Summary of up-regulated contigs

Up-regulated contigs in response to *B. bassiana* challenge		
Annotation	Fold-change	Taxon
Amidohydrolase 2	3.43	Bacteria
Peroxiredoxin-mitochondrial	2.81	Termite
Glutathione S-transferase (GST)	5.10	Termite
Ferritin	2.85	Termite
10 kDa Heat shock protein	3.40	Termite
Cytochrome b-c1 subunit 10	3.91	Termite
Cytochrome b-c1 subunit 7	3.04	Termite
Cytochrome b-c1 subunit 9	4.30	Termite
Cytochrome c	2.83	Termite
Cytochrome c oxidase subunit 6B	2.98	Termite
Cytochrome c oxidase subunit 6C	3.13	Termite
Cytochrome c oxidase subunit 7C	2.93	Termite
NADH dehydrogenase 1 alpha subunit	3.60	Termite
3'–5' exonuclease	2.82	Protist
3'–5' exonuclease/DNA Polymerase I	3.25	Protist
Ca2+/calmodulin dependent kinase II (CAMKII)	4.15	Protist
Mitogen-activated protein kinase 1 (MAPK1)	3.49	Protist

Significantly up-regulated contigs at 48-h post-inoculation with *B. bassiana*. Annotation and taxon based on RBH to the custom termite consortium database. Fold-change represents Log_2_ CPM Treatment/CPM Control as calculated by edgeR

Up-regulated protist contigs also have annotations associated with oxidative stress, as well as general stress responses. Of note, two up-regulated protist contigs were annotated as 3’–5’ exonucleases/DNA Pol I, which is associated with oxidative stress-linked DNA repair. The signal cascade initiators Ca^2+^/CAMKII and MAPK1 were also up-regulated, and have links to oxidative stress response. The only bacterial contig in the up-regulated category with a named annotation is an amidohydrolase family 2 member. These enzymes have a wide-variety of catalytic capabilities, including activity against fungal metabolites.

### Beauveria bassiana challenge results in oxidative stress

**Fig. 4:**
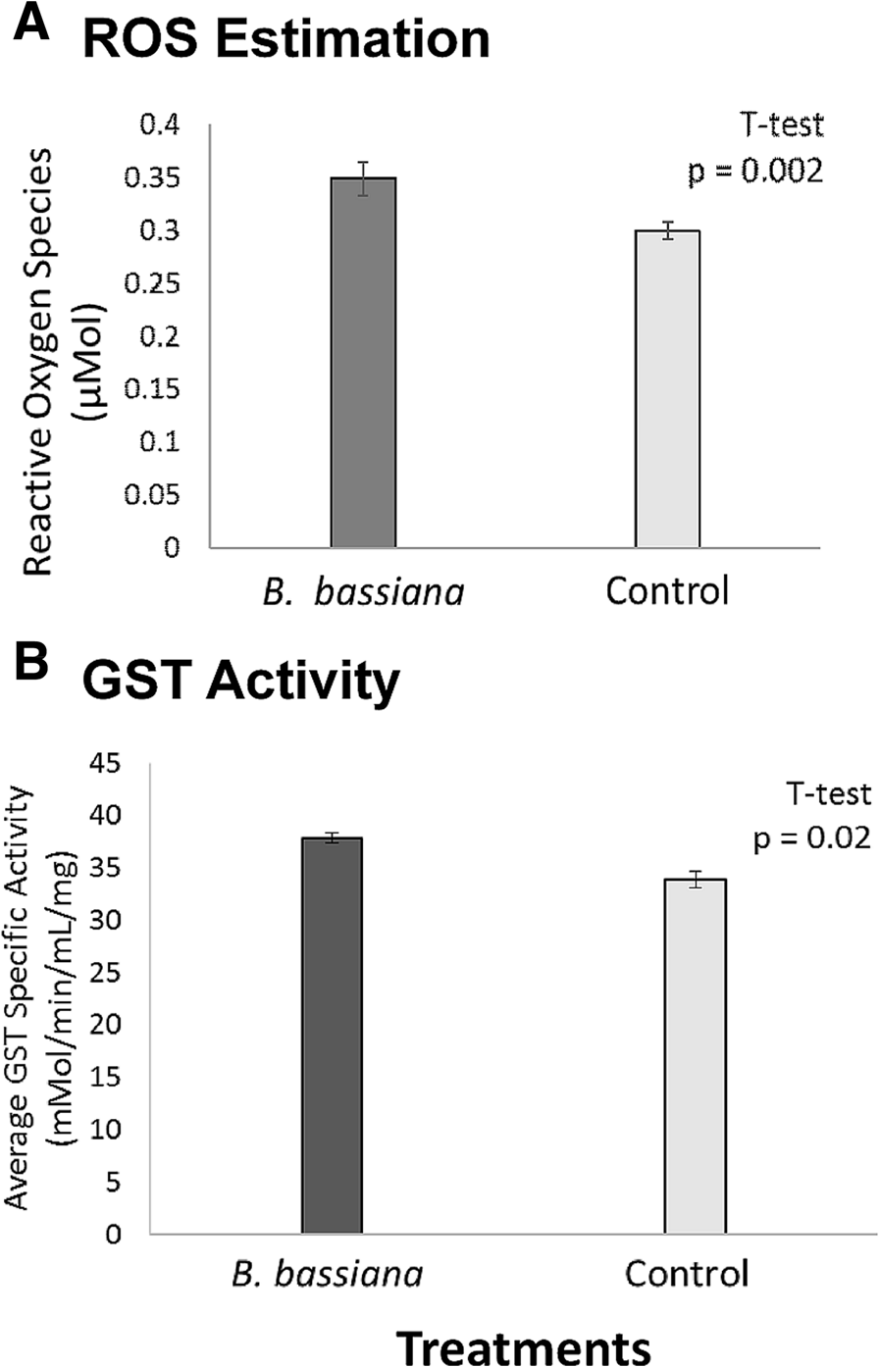
Results of post-hoc experiments to estimate oxidative stress. **a** Detection of reactive oxygen species (ROS) following *B. bassiana* challenge. Bars represent measured reactive oxygen species in *B. bassiana* (dark bar) and no treatment control (light bar) termite worker guts. **b** Detection of glutathione S-transferase (GST) activity following *B. bassiana* challenge. Bars represent measured GST specific activity in *B. bassiana* treatments (dark bar) and negative controls (light bar) for termite worker guts normalized to blanks that received no reduced glutathione. Error bars represent SEM

In addition to the up-regulation of genes related to oxidative stress response, following 48-h challenge with *B. bassiana*, termite guts had increased ROS present (Fig. 4a). Additionally, GST activity is significantly higher by 1.15X in pathogen challenged guts than control guts (Fig. 4b). While the origin of ROS and antioxidant/detoxification activity cannot be identified using this method, it can still be concluded that the termite gut is under oxidative stress 48-h after inoculation with *B. bassiana*.

### Symbiont reduction results in increased *B. bassiana* susceptibility and reduced amidohydrolase induction by *B. bassiana*

**Fig. 5:**
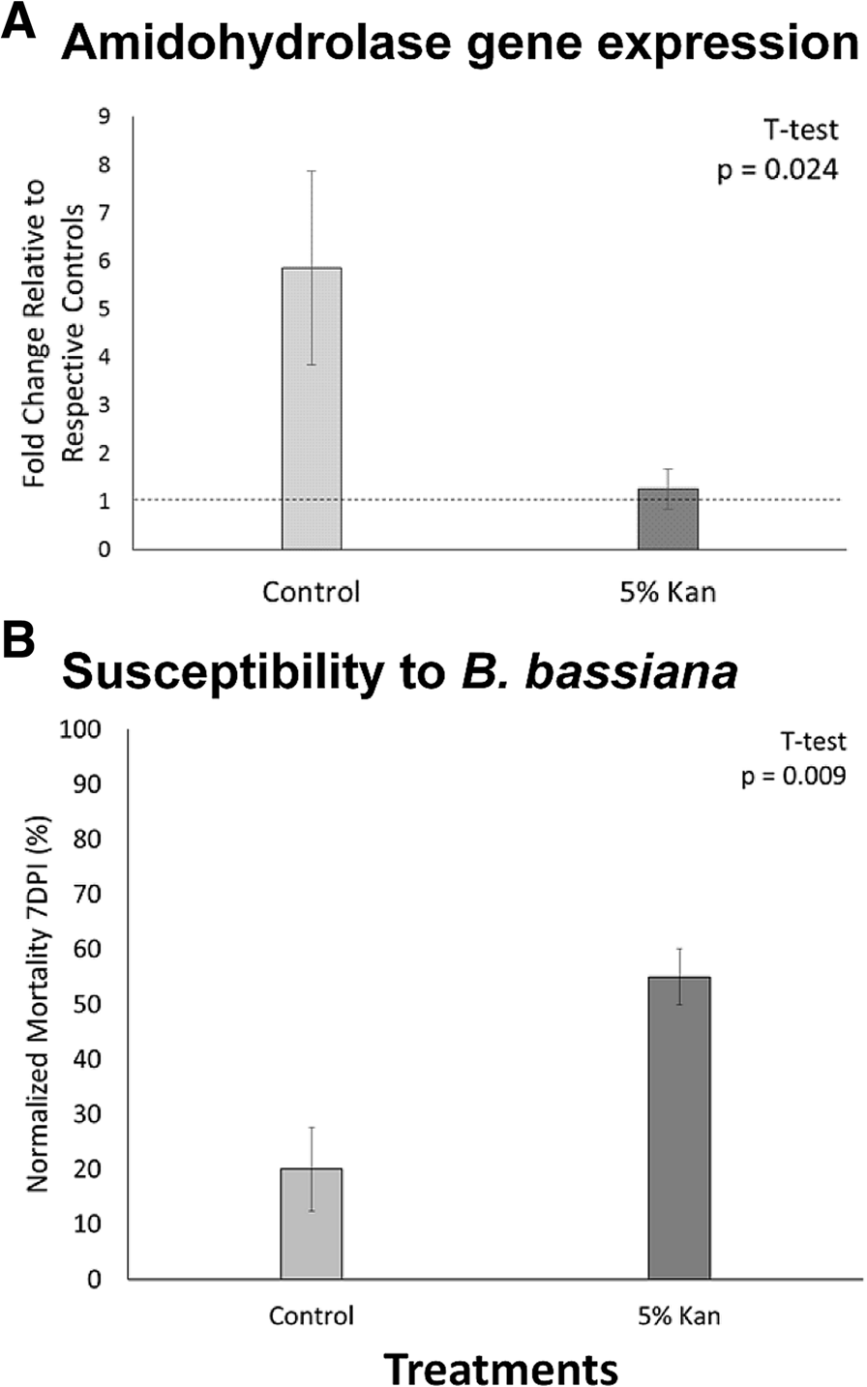
Results of post-hoc experiments to investigate bacterial Amidohydrolase 2 gene expression and *Beauveria bassiana* susceptibility following treatment with the antimicrobial drug kanamycin (Kan). **a** Relative Amidohydrolase 2 expression following *B. bassiana* pathogen challenge with and without 48-h 5 % kan treatment. Control group represents the fold-change in Amidohydrolase 2 gene expression in pathogen challenged termites relative to unchallenged, water-treated controls. The 5 % Kan group represents the fold change in Amidohydrolase 2 gene expression in 5 % kan treated, pathogen challenged termites relative to an unchallenged, kanamycin-treated controls. **b** Normalized mortality at 7-days post inoculation with *B. bassiana*, following either water (control) or 5 % kan treatments. Bars represent normalized mortality to the respective controls of each group, i.e. water treated, unchallenged controls or 5 % kan treated, unchallenged controls. Error bars represent standard error of the mean across 3 biological replicates

Antibiotic treatment also impacted the expression of the amidohydrolase 2 gene, which was induced by *B. bassiana* challenge in the metatranscriptome dataset. Following treatment with kanamycin, an antimicrobial drug, amidohydrolase 2 gene expression was reduced ~5X in pathogen challenged termites compared to water treated controls (Fig. 5a). Finally, in agreement with the amidohydrolase result above, treatment with kanamycin resulted in a 3X increase in termite susceptibility to *B. bassiana* (Fig. 5b) underscoring the apparent relevance of the amidohydrolase gene as a mechanism of bacterial-mediated fungal defense.

## Discussion

### Ribo-depletion produces a quality metatranscriptome assembly

One of the goals of this research was to identify candidate genes facilitating symbiont-mediated fungal pathogen defense in *R. flavipes*. To do this, a unique transcriptome preparation and analysis approach was used that allowed for the ribodepletion of rRNA from total RNA rather than enriching (and potentially biasing) for mRNAs. A commercial library preparation kit was modified to efficiently deplete all total RNA samples of anticipated prokaryotic and eukaryotic rRNAs. This strategy resulted in low rRNA content in the sequenced libraries and yielded a robust assembly of over 2 million contigs, >30,000 of which were annotated through our annotation pipeline. The contig annotation pipeline took a conservative approach to identifying termite, bacterial, protist, and archaeal sequences from a custom termite consortium database built from publically available sequence data (Table 1). Though this pipeline likely misses novel transcripts and underestimates redundancies, this conservative approach produced contig annotations with reasonable confidence. Additionally, identifying 9730 best reciprocal hits, out of the 14,610 annotated genes in the *Z. nevadensis* OGS [37], from gut tissue of worker termites in a different taxonomic family of Isoptera, speaks to the quality of the assembly.

Contrastingly, the reference sequences for protist and archaeal symbionts proved to be inadequate for the purposes of this study. With only 248 and 174 RBH respectively, undoubtedly important protist and archaeal contributions to this system have been under sampled, both generally and in reference to fungal defense specifically. For instance, the absence of candidate protist-derived β-1, 3-glucanases, described to play a role in *Metarhizium anisopliae* neutralization [31], is one apparent consequence of this conservative strategy. However, the termite and bacterial libraries are comparatively more complete. From these annotated contigs a more complete picture of bacterial contributions to their termite host and the gut consortium as a whole can be ascertained. Compared to previous metatranscriptomic efforts using mRNA enrichment in this system, the present assembly has identified many more bacterial contributions, further emphasizing the advantages of a ribodepletive strategy [30, 36]. Similar to studies in higher termites [14, 29], which lack protist symbionts, this assembly showcases a diversity of carbohydrate active genes including over 200 glycosyl hydrolases of bacterial origin. As described previously, bacterial symbionts appear to possess a complete amino acid biosynthetic toolkit (Table 3) [17, 23, 24, 39]. Additionally, the presence of nitrogen metabolism genes like nitrogenases, nitroreductases, and ureases corroborates the importance of bacterial symbionts for nitrogen recycling and fixation in the lower termite gut [15, 16, 19, 43, 46]. As with other recent studies in lower termites, this study corroborates that bacteria in the *R. flavipes* gut express a diversity of carbohydrate metabolism transcripts [12, 36, 39, 47]. These annotations include cellulases and hemicellulases, glycosyl transferases, carboxylesterases, and polysaccharide lyases, and they appear to be both complementary and redundant to those encoded by the host termite (Table 3). Once again, this suggests that bacteria of lower termites like *R. flavipes* play an active role in wood digestion [12, 28, 39, 47] and fails to support the idea that carbohydrate metabolism is completely restricted to flagellate/protist digestive vacuoles precluding bacterial catabolism [2].

In addition to anabolic and catabolic potential, the termite gut metatranscriptome is rich in bacterial transporters. These transporters, particularly ABC transporters, shed light on a practical aspect of the termite gut symbiosis. With a total of 787 annotated bacteria-derived transporters, including those responsible for transporting all types of organic molecules and metal ions, the idea of a hypercollaborative *R. flavipes* gut environment with influx and efflux of all types of compounds between members of the consortium is supported. ABC transporters have been noted as playing important roles in other insect-microbe relationships, particularly where metabolic partitioning is involved [25, 35, 45, 49]. Taken together, the complementary nature of the bacterial and termite contigs found in the gut metatranscriptome provides the foundation for a more complete view of this tripartite symbiosis.

### *B. bassiana* challenge results in oxidative stress

Findings of this study indicate that, 48 h post inoculation with *B. bassiana* conidia, the termite gut experiences general oxidative stress. ROS estimation indicates a significant increase in oxidative stress in pathogen challenged guts (Fig. 4a). Additionally, up-regulation in host antioxidant enzyme-coding genes for peroxiredoxin and GST, are corroborated at the protein-level with increases in GST enzyme activity (Table 5, Fig. 4b). One question this result raises is, *what is the origin of the observed ROS?* Presumably, any or all members of the consortium or the pathogen could produce free radicals. One possibility suggested by the transcriptome data, however, is that the up-regulation of OXPHOS complexes I, III, and IV without corresponding up-regulation of ATP synthase may result in uncoupling-related proton leakage, thus resulting in increased ROS [20]. Production of ROS in response to pathogen challenge is a common defense strategy in eukaryotes, and coupled with more traditional immune-associated pathways [3, 44], may serve as a mechanism for endogenous termite anti-fungal defense.

In line with the increased oxidative stress that was observed, many up-regulated symbiont contigs are also associated with response to this type of damage. Two protist transcripts encoding signaling kinases, CAMKII and MAPK1, are up-regulated and may be involved in triggering cascades responsible for coordinating stress responses like oxidative stress and pathogen challenge [5, 44]. Additionally, two 3’–5’exonuclease genes are also up-regulated. These genes encode enzymes like DNA Pol I, which is responsible for DNA repair and has been shown to be responsive to oxidative stress [18].

### Amidohydrolase 2, a candidate symbiont-mediated anti-fungal response mechanism

The primary aim of this project was to identify new candidate mechanisms of symbiont-mediated anti-fungal defense. The specific hypothesis tested was that bacteria collaborate with the rest of the holobiont to combat invaders. An ideal candidate gene should be up-regulated in response to pathogen presence and its product possess putative functions which might contribute to defense. Following these criteria, our dataset contained just one candidate: *amidohydrolase 2*. This amidohydrolase is a bacterial gene up-regulated 3.4X 48-h post-inoculation with *B. bassiana.* Amidohydrolases are a large family of diverse enzymes which are catalytically promiscuous [34]. These activities include hydrolysis, isomerization, and decarboxylation of diverse substrates [34]. Amidohydrolases are found across domains of life and are particularly of note in bacteria due to their role in antibiotic resistance [34]. Beta-lactamases, enzyme class 3.5.2.6, catalyze the deactivation of beta-lactam antibiotics like penicillin [4]. While *B. bassiana* does not produce penicillins, it is known to produce ooconidiain which it uses to evade insect anti-fungal defenses like PPO and antifungal peptides [13]. If this amidohydrolase is capable of hydrolyzing the quinone ooconidiain, this could contribute significantly to *B. bassiana* defense in the termite gut.

In post-hoc experiments, we found that antibiotic treatment with 5 % kanamycin both mitigates the *B. bassiana*-mediated induction of amidohydrolase 2 and results in a 3-fold increase in termite worker susceptibility to *B. bassiana* (Fig. 5). Previously, kanamycin has been shown to reduce both bacterial and protist symbiont populations in *R. flavipes* worker guts [28]. These findings provide strong evidence to confirm the importance of symbiont-mediated protection against *B. bassiana* and further implicate amidohydrolase 2 as a potential, bacteria-contributed mechanism of protection.

In addition to amidohydrolase, there are 15 symbiont (6 protist and 9 bacterial) contigs that exhibit significant fold-changes in response to *B. bassiana* pathogen challenge. While these genes have no known function, the possibility that they possess important anti-fungal properties cannot be excluded. Unfortunately, this possibility cannot be ascertained at the present time given the current information available in public repositories like NBCI’s RefSeq and Uniprot’s ID mapping databases.

Finally, there remains the possibility that key symbiont-derived enzymes associated with anti-fungal defense are expressed earlier or later in the infection timeline. The 48-h time-point was used in the current study based on a previous study that showed fungal pathogen-induced changes to gut gene expression [33]. However, that study was done with a different fungus using vastly different microarray technology, and as such 48-h post-inoculation may not be the optimum time-point for such observations. Future studies on this topic may thus wish to consider different time points post-inoculation.

## Conclusion

In sum, this study has provided the most complete gut metatranscriptome from *R. flavipes* to date, especially in regard to bacterial contributions. Our findings shed light on the physiological collaboration in the termite gut consortium with regard to biosynthesis, catabolism, and transport of major organic molecules and ions. Notably, these data corroborate previous findings suggesting that bacteria possess the potential to play direct roles in nitrogen fixation, amino acid biosynthesis, and lignocellulose digestion.

**Fig. 6:**
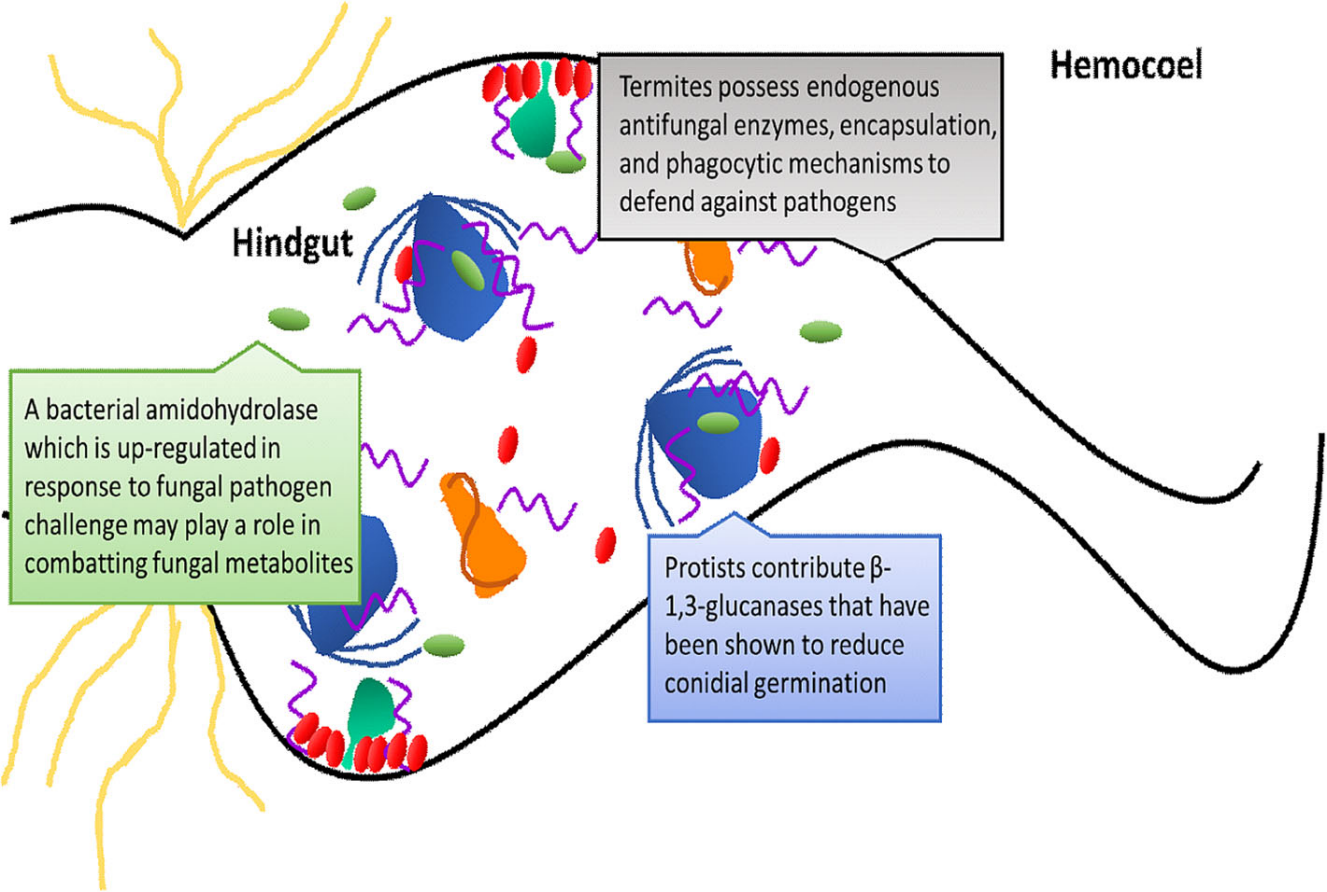
A proposed model of collaborative immune physiology. Protists, bacteria, and the termite host all contribute to neutralizing fungal invaders within the termite hindgut

Additionally, this study reveals a potentially novel mechanism for bacterial-mediated anti-fungal defense by means of amidohydrolase 2 enzyme action. The transcript for this gene is up-regulated 3.4-fold 48-h post-inoculation with *B. bassiana* and, based on the diversity in this enzyme class, may catabolize fungal metabolites which inhibit insect immune responses and have antibiotic activity. This possibility is further supported by independent post-hoc studies showing concomitant reductions in amidohydrolase gene expression along with increases in *B. bassiana* susceptibility after treatment with the antimicrobial drug kanamycin (Fig. 5). Coupled with previous findings of protist-derived anti-fungal defenses, this proposed model of antifungal defense highlights the collaborative nature of immune physiology within the termite holobiotic system (Fig. 6).

## Additional file

Additional file 1:
**Table S1.** Custom ribo-depletion primers developed in conjunction with NuGen to deplete the anticipated eukaryotic members of the termite holobiont and the fungal pathogen in treatment samples. **Table S2.** Primers used in this study for qPCR validation. Primers were designed using Primer3 program (http://frodo.wi.mit.edu/) or were adapted from previous studies. (DOCX 19 kb)

## Abbreviations

ABC: ATP-binding cassette
CAMKII: Ca^2+^/calmodulin dependent kinase II
cDNA: Complimentary DNA
CDNB: 1-chloro-2,4-dinitrobenzene
CPM: Counts per million
CT: Cycle threshold
DNA Pol I: DNA polymerase I
GO: Gene ontology
GST: Glutathione S-transferase
MAPK1: Mitogen-activated protein kinase 1
mRNA: Messenger RNA
NCBI: National Center for Biotechnology Information
OGS: Official gene set
OXPHOS: Oxidative phosphorylation
PDA: Potato dextrose agar
PPO: Prophenoloxidase
PUGC: Purdue University genomics core
qPCR: Quantitative real-time polymerase chain reaction
RBH: Reciprocal best hit
ROS: Reactive oxygen species
rRNA: Ribosomal RNA
