# Supplementary material for: Metatranscriptome analysis reveals bacterial symbiont contributions to lower termite physiology and potential immune functions
Source: BMC Genomics. 2016 Oct 1;17:772. doi: 10.1186/s12864-016-3126-z (PMC5045658; doi:10.1186/s12864-016-3126-z)
Supplement: Additional file 1: — Table S1. Custom ribo-depletion primers developed in conjunction with NuGen to deplete the anticipated eukaryotic members of the termite holobiont and the fungal pathogen in treatment samples. Table S2. Primers used in this study for qPCR validation. Primers were designed using Primer3 program (http://frodo.wi.mit.edu/) or were adapted from previous studies. (DOCX 19 kb) [file 12864_2016_3126_MOESM1_ESM.docx]

Table S1. Custom ribo-depletion primers developed in conjunction with NuGen to deplete the anticipated eukaryotic members of the termite holobiont and the fungal pathogen in treatment samples.

| Primer Name | Primer Sequence |
| --- | --- |
| >1_EF363230\|_0 | ATGCATGTCTCAGTGCAAGC |
| >2_EF363230\|_70 | TGGTTCCTTAGATGGTGGACA |
| >3_EF363230\|_140 | GACGGAAGGGACGCTTTTAT |
| >4_EF363230\|_210 | GTTTGCCTTGGTGACTCTGAA |
| >5_EF363230\|_280 | CTGTCGATGGTAGGCTCTGC |
| >7_EF363230\|_420 | ACGGGGAGGTAGTGACGAA |
| >8_EF363230\|_490 | ATCCATTGGAGGGCAAGTCT |
| >9_EF363230\|_560 | AAGCTGTTGCGGTTAAAAAGC |
| >10_EF363230\|_630 | CCCGTCGGTGTTTAACTGG |
| >11_EF363230\|_700 | AGCGGTAACGTTCTCACACC |
| >12_EF363230\|_770 | CCGGCACGTTTACTTTGAAC |
| >13_EF363230\|_840 | TGAATACCGAGTGCATGGAA |
| >14_EF363230\|_910 | TCCCCGAGGTAATGATCAAA |
| >15_EF363230\|_980 | CGAAAGCATTTGCCAAGAAT |
| >16_EF363230\|_1050 | TTCGAAGGCGATCAGATACC |
| >17_EF363230\|_1120 | CCGAAGTTCCTCCGATGAC |
| >19_EF363230\|_1260 | CTCAACACGGGAAACCTCAC |
| >20_EF363230\|_1330 | GTGGAGCGATTTGTCTGGTT |
| >21_EF363230\|_1400 | AGTCGCATCCGGTATCCTTT |
| >22_EF363230\|_1470 | CTTCTAGCCGCACGAGATTG |
| >23_EF363230\|_1540 | CTGAAGGAATCAGCGTGTCC |
| >24_EF363230\|_1610 | TGAACCTCCTTCGTGCTAGG |
| >26_EF363230\|_1750 | CTTCGATTCCACTGGGAAGA |
| >28_AB032204\|_0 | GCCATGCAAGTGCTAGTTCA |
| >29_AB032204\|_70 | ACACGCTCAGAACCCATTTG |
| >30_AB032204\|_140 | GACATGACCTTATAGGCGTACCA |
| >31_AB032204\|_210 | TGTAGGCTATCACGGGTAACG |
| >33_AB032204\|_350 | CAAGATCGGCGGATAGGTT |
| >35_AB032204\|_490 | GCTAATCGCGTTCCAATGTT |
| >36_AB032204\|_560 | CTGAATGACTCAGCACGGTATG |
| >38_AB032204\|_700 | GGATGCGAAAGCGTTTACCT |
| >39_AB032204\|_770 | GACGGAGCGTTGTGCTATTG |
| >40_AB032204\|_840 | AGGCCTATTGGGGAACTACG |
| >41_AB032204\|_910 | TCAACGCGGAGAAACTTACC |
| >42_AB032204\|_980 | TGACTGACCGGCTAAAGACC |
| >43_AB032204\|_1050 | GGTGATTCGTGAAGCGATTT |
| >45_AB032204\|_1190 | GCTGCACGCGTTCTACAAT |
| >46_AB032204\|_1260 | CCTGAGAGGGTTTGCTACTCC |
| >47_AB032204\|_1330 | TCCCTTGTAAGCGTACGTCAA |
| >49_AB032205\|_70 | ACCCGCAAGGGTACTAAAGG |
| >50_AB032205\|_140 | GTTAAGGTGAGGACGTGACCA |
| >51_AB032205\|_210 | CCTATCAGCTTGTCCGCAGT |
| >52_AB032205\|_280 | TGAGAGACAGCGGCTATTCC |
| >53_AB032205\|_350 | CAACGAAGGAGGTGGTAACG |
| >54_AB032205\|_420 | TGTGGGGTAACCTAGGAGAGG |
| >55_AB032205\|_490 | AAGCTCGGATAGAGCGTTCA |
| >56_AB032205\|_560 | CGTGACCAAATCAGAATGCTT |
| >57_AB032205\|_630 | TCAGATCAAAGAGAGCCATCG |
| >58_AB032205\|_700 | GAAACGAATGCGAAAGCATC |
| >59_AB032205\|_770 | GGGTAGTTGCGGCCTTAAAC |
| >60_AB032205\|_840 | AGCGAAAGCTTGAGTCGTTG |
| >62_AB032205\|_980 | TGATTTTTGGTGGTGCATGG |
| >63_AB032205\|_1050 | CGTGGATTGATCTGTCATGC |
| >64_AB032205\|_1120 | GTTTAAGCAGGCGGAAGAGG |
| >66_AB032205\|_1260 | AGCGTAGTTGGGATTGACGA |
| >67_AB032205\|_1330 | CAACATTGCGCGTTGAATAC |
| >68_AB032206.1\|_0 | AAGGAAGCACACTTCGGTCA |
| >69_AB032206.1\|_70 | TGGTTTTAAATGGATAGCAGAGGT |
| >70_AB032206.1\|_140 | ATGCGATTGTTTCTCCAGAAGT |
| >72_AB032206.1\|_280 | CCATATCTACGGGTAGCAGCA |
| >73_AB032206.1\|_350 | TCGGAGGAGGTAATGACCAG |
| >74_AB032206.1\|_420 | CGTCGTGAAATCTAGCAGAGG |
| >75_AB032206.1\|_490 | AACGCCCGTAGTCTGAACTG |
| >76_AB032206.1\|_560 | TCCATTCGTTCACTGCGAAT |
| >77_AB032206.1\|_630 | TGAGAATCATCGGGGGTAGA |
| >79_AB032206.1\|_770 | GATCAAGGGCGAGAGTAGGAG |
| >80_AB032206.1\|_840 | CAAAATAGCAGTTTCGCAGGA |
| >81_AB032206.1\|_910 | TTGAAGGAATTGACGGAAGG |
| >83_AB032206.1\|_1050 | GGTTGACCTGTCTAGCGTTGA |
| >84_AB032206.1\|_1190 | TCCGTGATGTCCTTTAGATGC |
| >85_AB032206.1\|_1260 | CGACAGGGCACGCTACTCT |
| >86_AB032206.1\|_1330 | CCAGGAATCCCTTGTAAATGTG |
| >88_AY137594\|_70 | ACCGTCCATGCATGCTTTA |
| >90_AY137594\|_210 | CAGGGTTCGATACCGGAGAG |
| >91_AY137594\|_280 | ATGAGAAATGGCGACCATCA |
| >92_AY137594\|_350 | CTTCGGTTCGACAATTGGAA |
| >95_AY137594\|_560 | TCGGTTGAGGGTTGATTCAT |
| >98_AY137594\|_770 | TCGCAGCTGAACACATTAGC |
| >99_AY137594\|_840 | TGTAGTTTCCCGGCTTTGTC |
| >101_AY137594\|_980 | GACTGTCGGGGGCATTAGTAT |
| >104_AY137594\|_1190 | GCGTTAAGTTTTCGGGTTCA |
| >107_AY137594\|_1400 | TGGTGCATGGTCGTTCTTAGT |
| >108_AY137594\|_1470 | AAAGTGTTGTGGCATGGTCA |
| >112_AY137594\|_1750 | TTACGTCCCTGCCCTTTGTA |
| >114_AY137592\|_0 | GTGCGTAAAAGCCTGACTGC |
| >115_AY137592\|_70 | GGAAGGGCCGTGTTTATTAGA |
| >116_AY137592\|_140 | AATCGTCGTATCGACCTTGTG |
| >120_AY137592\|_420 | CCGCGGTAATTCCAGCTCTA |
| >122_AY137592\|_560 | CTCATTGGCGCTGAGATTG |
| >123_AY137592\|_630 | TGGTCCACTTTGGTGTGGTT |
| >125_AY137592\|_770 | GGACATCAGTGGGGTACTCG |
| >126_AY137592\|_840 | GATGGCAGCTTGTTTGGTG |
| >127_AY137592\|_910 | ATGCATCCGTTTTGTTGGTT |
| >130_AY137592\|_1120 | GGATTGGGGGTTGACCTTTA |
| >139_AY137592\|_1750 | CGCTCCTACCGATGAATGAT |
| >148_AY137593\|_490 | ATGGAGCTTTTCGTCCCTGT |
| >149_AY137593\|_560 | TTTTTCGTCGTGGGTTGATT |
| >153_AY137593\|_840 | TCTTTGTTGGGCAGGTTGTT |
| >160_AY137593\|_1330 | GTTCGGACACGGTGAGGAT |
| >162_AY137593\|_1470 | GGGGTGGTTACCGTTCCTTA |
| >163_AY137593\|_1540 | GGGGGAAGTGAGGCAATAAC |
| >164_AY137593\|_1610 | GTTTGTCCTGGCTTGGAAGA |
| >165_AY137593\|_1680 | GTGAACGCGGAATATCTCGTA |
| >167_AY137593\|_1820 | CCGCGACAAAAGAGCAGTA |
| >168_AY137595\|_0 | CGCAAACGCCCTACTATGTG |
| >170_AY137595\|_140 | TCGTATCGACTTTTGTCGATTTT |
| >171_AY137595\|_210 | CCTGACGTTAAGGTCGTGTCTT |
| >172_AY137595\|_280 | GGCCACAATTGCTAAGGTGA |
| >176_AY137595\|_560 | GGGTTTGCTGTCTGGATGTC |
| >180_AY137595\|_840 | AGCTCGAAAGGGTTAATTGGTT |
| >181_AY137595\|_910 | CGGGAGGAGAGGGGTAGAT |
| >183_AY137595\|_1050 | TGCGAAAGCATTCTTCAAGG |
| >185_AY137595\|_1190 | TTGGGGGTTGACTCAAATACA |
| >191_AY137595\|_1610 | TGAACCGCACGCGTACTACT |
| >192_AY137595\|_1680 | CTGGGGATCGGTTTTTGTAAT |
| >195_AF244903\|_0 | CCTGGTTGATCCTGCCAGT |
| >197_AF244903\|_140 | ACATGCGCAAAATCCTGACT |
| >198_AF244903\|_210 | GTCCTGCACCTTGGTGATTC |
| >201_AF244903\|_420 | CCAACACACTGGGGAGGTAG |
| >202_AF244903\|_490 | TGTAATTGGAATGGTCCGAACT |
| >204_AF244903\|_630 | CCAGGGGACGGATATTTCTC |
| >205_AF244903\|_700 | GGATTCGGAGCCTTTACTTTG |
| >207_AF244903\|_840 | GCATTTGTATTGCGTCGTCA |
| >210_AF244903\|_1050 | CAAACGACCCTTTCAGCACT |
| >214_AF244903\|_1330 | CGAGACCTCAGCCTGCTAAC |
| >215_AF244903\|_1400 | TGGTGGAAGTTTGAGGCAAT |
| >219_AF244903\|_1680 | GTGGTTTGGTGGGAAGAACA |
| >220_AF244903\|_1750 | CCGTAGGTGAACCTGCAGAA |
| >224_AF244904\|_210 | ACGCGTCCTTTGGTGATTC |
| >225_AF244904\|_280 | GGCTTGCCTTCGATGTATCA |
| >226_AF244904\|_350 | TTTTGACGGGTAACGGAGAA |
| >230_AF244904\|_630 | GGAGTTATGCTGCGGTCATT |
| >233_AF244904\|_840 | TTTTGTTGGTTTTCGAGACGA |
| >242_AF244904\|_1470 | CCGTCAACAAGCTCATCCTG |
| >245_AF244904\|_1680 | TCCGATGAAATGCTTGGACT |
| >249_AF244905\|_140 | CGGTGTAGTCGGCGCTTA |
| >250_AF244905\|_210 | CCGCAAGGTTTCAGATTTGT |
| >251_AF244905\|_280 | CTCGTATCTCGGCGATTCAT |
| >254_AF244905\|_490 | CATGGCTTGTAATTGGAATGG |
| >256_AF244905\|_630 | CGTAGTTGGATTTCGGAGGA |
| >257_AF244905\|_700 | CTGAGTGGGGGTAGGGATTT |
| >258_AF244905\|_770 | AAGCAGGCTTAACGCACCT |
| >259_AF244905\|_840 | TTGTTGGTTTATAGGATTGGTCGT |
| >262_AF244905\|_1050 | GGGATCGGAGGGTGTTAAAT |
| >267_AF244905\|_1400 | ACATGGGGGAAGTTCTAGGC |
| >268_AF244905\|_1470 | TGATGCCCTTAGATGTTCTGG |
| >269_AF244905\|_1540 | TCGTTATCGTGATGGGGATT |
| >272_AF244905\|_1750 | GGTTGTGATCGGATGAGAAGA |
| >273_AF244906\|_0 | TGACACTGCGAAAAGCTCCT |
| >274_AF244906\|_140 | CGAAAACCAGCGCTCTATGT |
| >275_AF244906\|_210 | CTCGGTCGATTTTTCATTCAA |
| >281_AF244906\|_630 | TTGATACGGGTTTGCTGGTC |
| >282_AF244906\|_700 | CAGGGATTGCTTCGTCAATTA |
| >283_AF244906\|_770 | GGTCCGGAAGGTTTACTTTGA |
| >285_AF244906\|_910 | TCCTGTCGAGTGTTGGCTATC |
| >286_AF244906\|_980 | TTGCAGGGAGACAGGAATGT |
| >288_AF244906\|_1120 | CGTTTATCATGGGCGTTTTC |
| >294_AF244906\|_1540 | CGAAAGTGTCGTGGTGTTTTT |
| >296_AF244906\|_1680 | CGCCTCAATGAGTTTGTGGT |
| >298_AF244906\|_1820 | GTCCCTGCCCTTGTACACAC |
| >299_AF244906\|_1890 | CAGGTGAAATGCTCGGATAGA |
| >300_AF244906\|_1960 | GGAAGGAGAAGTCGTAACAAGGT |
| >301_AF280633\|_0 | TACAGCGAAACTGCGAATGG |
| >302_AF280633\|_70 | TGGATAACCGTGGTAATTCTAGAGC |
| >303_AF280633\|_140 | CGACTTCGGAAGGGAGGTAT |
| >305_AF280633\|_280 | GGTATTGGCCAAACATGGTC |
| >306_AF280633\|_350 | TACATCCAAGGAAGGCAGCA |
| >307_AF280633\|_420 | AGGGCTCTTTTGGGTCTTGT |
| >310_AF280633\|_630 | CCTTTCCCTCTGTGGAACCT |
| >311_AF280633\|_700 | TGCTCGAATACATTAGCATGGA |
| >312_AF280633\|_770 | TTTCTAGGACCGCCGTAATG |
| >315_AF280633\|_980 | TCGGCACCTTACGAGAAATC |
| >317_AF280633\|_1120 | GCAGTAGCTCTGCTCCCAAA |
| >318_AF280633\|_1190 | GGCGAACCAAAGTGCTAGTCT |
| >319_AF280633\|_1260 | TTCTAAAACCAGTGTCACCAAGC |
| >320_AF280633\|_1330 | ACGGGGAAGGTTCAGAGACT |
| >321_AF280633\|_1400 | GCTCGCTCACACACTGCTTA |
| >322_AF280633\|_1470 | CGGATTGGCAAGCTCAAATA |
| >325_AF280633\|_1680 | GCCTGTATTGCTTTGGCAGT |
| >327_AF280633\|_1820 | ATCTTGTGAAACTCCGTCGTG |
| >328_AF280633\|_1890 | CAAGTCATCAGCTTGCGTTG |
| >329_AF280633\|_1960 | ACCGATTGAATGGCTCAGTG |
| >330_AF280633\|_2030 | CCGGAAAGCTCTCCAAACTC |

Table S2. Primers used in this study for qPCR validation. Primers were designed using Primer3 program (http://frodo.wi.mit.edu/) or were adapted from previous studies.

| Primer Name | Sequence (5'-3') |
| --- | --- |
| 14-3-3-F | GCGGCAGTTATTGAAAAGGA |
| 14-3-3-R | GCGATAGTAATCGGCCTTCA |
| Actin 5C-1- F | TCTGGTAGGACCACTGGTAT |
| Actin 5C-1-R | GTATCCACGCTCCGTCAAA |
| Aldo-keto reductase-F | TGCTAGTCGTTCAGCATCCA |
| Aldo-keto reductase-R | GAATTAAGCAGGCCCAGACA |
| Amidohydrolase 2-F | GGTCGGGCTTAATTGTGAGA |
| Amidohydrolase 2-R | CAGAGAACAAACACGGCAAA |
| Calmodulin-F | CAGCTGACTGAGGAACAGAT |
| Calmodulin-R | CGCCATCATTGTCAGGAAT |
| CalpainB-F | GACCGAGTGCTGACAAAACA |
| CalpainB-R | CTTTTGTACGTGTGCCATCG |
| Cell-1 qPCR-F | TCACAAGCAAGCAGGCATAC |
| Cell-1 qPCR-R | ATGAGAGCAGAATTGGCAGC |
| Cytochrome p450 9e2-F | TGTCCACATAACGGCACAGT |
| Cytochrome p450 9e2-R | TCTGTGCTTTGCCTCACATC |
| Ferritin2-F | TGAGGAGTGTTCGTGGTGAG |
| Ferritin2-R | GGCATCAATCGCTCCATATT |
| GHF-2 qPCR-F | GCACAACCGCAGTTATGGTC |
| GHF-2 qPCR-R | GCCCCAGATCGTGATGAAGA |
| Hypoxia Up-regulated-F | ATGTTTTGGTGAGGCAGACC |
| Hypoxia Up-regulated-R | CACTGGGCACTAAAGCAGGT |
| Nitroreductase-F | GCTCTCGTTCCTGCGTTATC |
| Nitroreductase-R | GGATTTAGGATTGGGCAACA |
